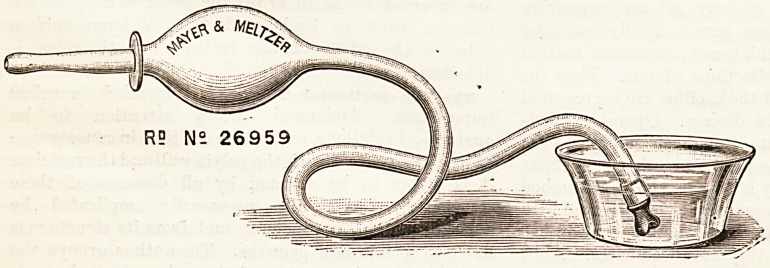# New Appliances and Things Medical

**Published:** 1896-12-05

**Authors:** 


### NEW APPLIANCES AND THINGS MEDICAL.

[We shall be glad to receive, at our Office, 28 & 29, Southampton Street, Strand, London, W.C., from the manufacturers, specimens of all new preparations and appliances which may be brought out from time to time.]

### AUTO ENEMA.

(Mayer and Meltzer, 71, Great Portland Street, London, W.)

This new enema strikes us as being particularly ingenious and useful. The bulb is placed close up to the nozzle so that it can be used as a handle, leaving the other hand free for any purpose that may be necessary. For the self-administration of enemata, this device is of the greatest value, and the extra length of the afferent tube will enable this operation to be carried out with a maximum of comfort.

### CONDENSED MILK, GOLD SEAL BRAND.

(New York Condensed Milk Company, New York.)

We have received a sample of the above condensed milk, and find it of excellent quality, with full percentage of cream and albuminous contents. Not only can we recommend it for general household purposes, such as for making coffee, cocoa, and chocolate, but also as a supplementary food for infants. There can be no doubt that judiciously used a good condensed milk, with full percentage of cream, will often enable a delicate child to tide over the difficulties attendant on deficient powers of digestion. In cases where failure is recorded it will generally be found that the condensed milk is inferior in quality and deficient in cream, misfortunes which will not occur if the "Gold Seal Brand" is used.

## Figures and Tables

**Figure f1:**